# The complete chloroplast genome sequence of *Lilium speciosum* Var. *gloriosoides*, an important breeding parent

**DOI:** 10.1080/23802359.2019.1696245

**Published:** 2019-12-09

**Authors:** Yixin Liu, Jie Huang, The Su Moe, Mohammad Sayyar Khan, Jing Xue, Xiuhai Zhang, Yunpeng Du

**Affiliations:** a Beijing Key Laboratory of Agricultural Genetic Resources and Biotechnology, Beijing Functional Flower Engineering Technology Research Center, Beijing Agro-Biotechnology Research Center, Beijing Academy of Agriculture and Forestry Sciences, Beijing, China;; b Division for Achievements Transformation and Promotion, Beijing Academy of Agriculture and Forestry Sciences, Beijing, China;; c Biotechnology Research Department, Ministry of Education, Mandalay Division, Pharmaceutical Research Laboratory, Kyaukse, Myanmar;; d Genomics and Bioinformatics Laboratory, Institute of Biotechnology and Genetic Engineering (IBGE), Faculty of Crop Production Sciences, University of Agriculture, Peshawar, Pakistan

**Keywords:** *Lilium speciosum* var. *gloriosoides*, complete chloroplast genome, next generation sequencing chloroplast, phylogenetic analysis

## Abstract

*Lilium speciosum* var. *gloriosoides* is an important breeding parent with high ornamental and edible value in worldwide. In this study, we reported a complete chloroplast genome of *L. speciosum* var. *gloriosoides*, which was *de novo* assembled using the next-generation sequencing data. The whole genome is 152,912 bp in length and includes one large single copy (LSC) region of 70,693 bp, one small single copy (SSC) region of 17,517 bp, and a pair of inverted repeat (IR) region of 26,539 bp. A total of 131 functional genes were encoded, consisting of 76 protein-coding genes, 36 transfer RNA genes, and eight ribosomal RNA genes. The overall AT content of the chloroplast genome is 63.00%. In the maximum likelihood, a strong phylogenetic signal showed that *L. speciosum* var. *gloriosoides* is a species of *Lilium*, which is the first major genome in section *Archelirion.*

*Lilium speciosum* var. *gloriosoides* is a perennial flower bulbs belonging to the section *Archelirion*, the genus *Lilium* (Liliaceae), which is characterized by bulb flattened subglobose, nectaries with red, fimbriate projections, and papillae on both surfaces (Liang and Tamura 2000). *Lilium speciosum* var. *gloriosoides* occurs in SW Japan (Kyushu, Shikoku); this species is only distributed in Anhui, Guangxi, Hunan, Taiwan, and Zhejiang of Province in China. It has high medicinal, ornamental, and horticultural values. Particularly, the bulbs can edible and medicinal value of antiviral activity (Chen et al. 2019). However, largely due to anthropogenic overharvesting and loss of natural habitat, the resources of *L. speciosum* var. *gloriosoides* have been dramatically decreased. Now, the species is distribution became shrinking in China, a good knowledge of its genomics datum would contribute to the effective management, the genetic background can be better understood, which is helpful in promoting the preservation of the species and great reference value for lily breeding. Simultaneously, this is useful for the conservation and exploitation of this valuable germplasm, and the molecular identification and phylogenetic study with other species in *Lilium*. In this study, we assembled and annotated the complete chloroplast genome of *L. speciosum* var. *gloriosoides* from Next Generation Sequencing data.

Samples of *L. speciosum* var. *gloriosoides* were collected from Tianmu Mountains (Geospatial coordinates: N:30°20′40″,E：119°25′30″) in Zhejiang, China, and DNA was stored at the herbarium of Institute of Botany, CAS (Herbarium number: BOP201947). Total genomic DNA was extracted from fresh leaves, according to the DNA secure Plant Kit (Aidlab). An Illumina paired-end library was prepared and used for Next Generation Sequencing on the HiSeq4000 Sequencing System at Novogene (http://www.novogene.com/index.php), Beijing, China. Then, top-quality reads were produced to map the whole genome using the program Sequencher 5.0 (Gene Codes Corporation, USA), with that of reported chloroplast genome of *Lilium* species (Kim and Kim 2013; Bi et al. 2016; Hwang et al. 2016; Du et al. 2016, 2017) as the reference. Assembled chloroplast genome was annotated using Dual Organellar GenoMe Annotator (http://dogma.ccbb.utexas.edu/) (Wyman et al. 2004). A physical map of the genome was drawn using OGDraw v1.2 (Lohse et al. 2013).

Whole chloroplast genome sequence of *L. speciosum* var. *gloriosoides* has been submitted to GenBank with the accession number MN509267. The genome is 152,912 bp in length and includes one large single copy (LSC) region of 70,693 bp, one small single copy (SSC) region of 17,517 bp, and two inverted repeat (IR) regions of 26,539 bp. It contains 131 genes, comprising 84 protein-coding genes, 36 transfer RNA, and eight ribosomal RNA genes. Among these genes, there are 20 genes that contain a single intron, which are *trn-KUUU*, *rps16*, *atpF*, *rpoC1*, *ycf3*, *trnL-CAA*, *trnV-UAC*, *clpP*, *petB*, *petD*, *rpl16*, *rpl2*, *ndhB*, *rps12*, *trnI-GAU*, *ndhA*, *trnA-UGC*, *trnI-GAU*, *ndhB*, *rpl2*, and two genes *ycf3* and *clpP* contained two introns. Out of these 19 genes, three genes (*trnA-UGC, trnI-GAU, ndhB, rpl2, rps12*) are partially located within in the IR regions. This genome composition is asymmetric (31.1% A, 18.9% C, 18.2% G, and 31.9% T) with an overall A + T content of 63%. The A + T content of the IR regions (57.6%) is obviously lower than those of the SSC (69.4%) and LSC (65.1%) regions, respectively.

Phylogenetic analysis was constructed using total chloroplast genome sequence of *L. speciosum* var. *gloriosoides* with nine published sequences in *Lilium* and outgroups of *Fritillaria hupehensis* and *Fritillaria taipaiensis* using maximum likelihood (ML) analyses (Figure 1). The ML tree was constructed at CIPRES (http://www.phylo.org) (Miller et al. 2010) using RAxML-HPC Black Box v.8.1.24 and branch support was estimated with 1000 bootstrap replicates. The ML tree results display 10 of 12 nodes that were supported by bootstrap values 100%, and the other two nodes by values < 50%, sect. *Archelirion* have no chloroplast genome sequenced, *L. speciosum* var. *gloriosoides* gathered on a single branch. The clustering of sect. *Sinomartagon*, sect. *Leucolirion*, and sect. *Martagon* is very clear and has a high support rate. The complete chloroplast genome can be subsequently utilized for genetic diversity, identifying species, taxonomy, and phylogenetic evolution studies for this species. Sect. *Leucolirion* is the original parent of Oriental *hybrid*. It is of great reference value to the cultivation breeding. It has important medicinal and ornamental value and can provide reference for other sect*. Archelirion* chloroplast genome sequencing. Simultaneously, it provides essential data for further study on the accurately identifying species, taxonomy, and phylogenetic resolution and evolution for the genus *Lilium*, and the available genome information also provides valuable insight into conservation and exploitation efforts for this endangered species.

**Figure 1.**
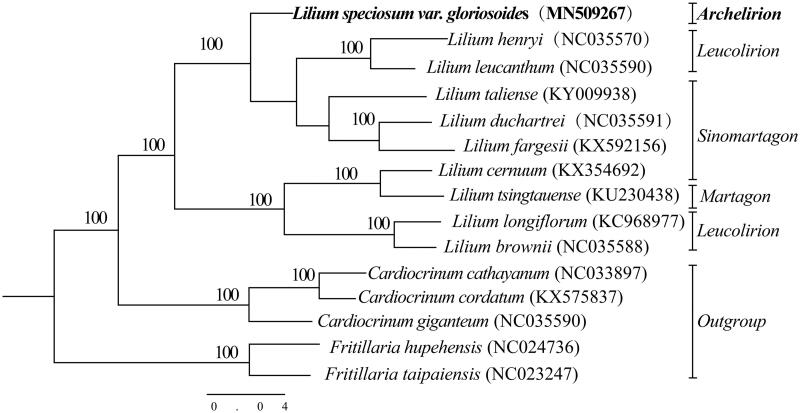
Phylogenetic relationships of 15 sequences in the genus *Lilium* with outgroups of two *Fritillaria* species constructed by whole chloroplast genome with the maximum likelihood (ML) analyses. The bootstrap values were based on 1000 replicates.
